# Studies on the health impact of *Agrimonia procera* in piglets

**DOI:** 10.1186/s12917-014-0210-y

**Published:** 2014-09-09

**Authors:** Tobias Gräber, Holger Kluge, Sebastian Granica, Gert Horn, Corinna Brandsch, Gabriele I Stangl

**Affiliations:** Institute of Agricultural and Nutritional Sciences, Martin Luther University Halle-Wittenberg, Von-Danckelmann-Platz 2, 06120 Halle (Saale), Germany; Department of Pharmacognosy and Molecular Basis of Phytotherapy, Medical University of Warsaw, Faculty of Pharmacy, Banacha St. 1, 02-097 Warsaw, Poland; Exsemine GmbH, Am Wehr 4, Salzatal, 06198 Germany

**Keywords:** Agrimonia procera, Polyphenol, Growth performance, Cytokine expression, Pig, Peripheral blood mononuclear cells

## Abstract

**Background:**

The weaning period is critical for stress-related diseases and infections. Currently, large amounts of therapeutic antimicrobials are used to treat infections in the livestock production, especially in piglets. Phytogenic feed additives could provide a useful alternative. We hypothesize, that components in agrimonia species which have been used successfully in humans to treat gastrointestinal infections could also improve the health of piglets*.* We investigated the effects of *Agrimonia procera* (AP) on the growth performance of piglets and cytokine expression in isolated porcine peripheral blood mononuclear cells (PBMC).

**Results:**

Here we show that piglets that received a diet with 0.56 g/kg AP for 6 weeks tended to ingest more food (+5.1%; P < 0.10), and were characterized by a higher nitrogen retention (+9.6%, P < 0.05) than the control group without AP treatment. Data from a second experiment reveal that piglets fed a diet with 0.87 g/kg AP for 6 weeks had an improved food conversion ratio (1.46 ± 0.04) compared to those that received none (1.54 ± 0.08) or 8.7 g/kg AP (1.60 ± 0.08) with their diets (P < 0.001). However, the food intake, daily weight gain and dry matter of feces were not affected by the AP treatment.

Treatment of PBMC for 1 and 6 h with AP extract (APE) reduced the mRNA abundance of tumor necrosis factor (TNF)α in cells challenged with lipopolysaccharides (LPS) but not in cells without LPS stimulation (P < 0.05). The lower mRNA expression of TNFα was accompanied by a trend towards a lower release of TNFα from these cells (P = 0.067). After the treatment of PBMC with APE for 6 h, the relative mRNA concentration of interleukin (IL)-1β declined (P < 0.05), whereas that of IL-10 remained unchanged. Treatment of LPS-challenged PBMC for 20 h with varying concentrations of APE did not reveal any effect on cytokine expression and TNFα release.

**Conclusions:**

The results indicate that low dosages of AP may improve the growth performance of piglets and seem to exert antiinflammatory effects in porcine immune cells challenged with LPS.

**Electronic supplementary material:**

The online version of this article (doi:10.1186/s12917-014-0210-y) contains supplementary material, which is available to authorized users.

## Background

For many decades it was a common practice to add sub-therapeutic amounts of antibiotics to the feed of pigs to control intestinal pathogens and to promote growth. However, the rising incidence of antibiotic-resistant bacterial infections prompted the European Union in 2006 to introduce a ban on growth-promoting antimicrobials (Regulation 1831/2003/EC). Nevertheless, the overall use of therapeutic antimicrobials to treat infections of farm livestock remained high. Throughout the fatting period, pigs received antimicrobials on average for 35 days, whereby most of the antimicrobials were administered to young animals [1]. Those data demonstrate that the weaning period is a critical period for stress-related diseases and infections during livestock production [1,2]. Stress factors such as weaning, pathogen exposure and littermate separation may induce reduced food intake, body weight loss and an increased susceptibility to gastrointestinal infections in these animals [3,4]. Botanicals and phytogenic feed additives are discussed as useful alternatives to the therapeutic use of antimicrobials because they are supposed to modulate the immune system and to improve performance of pigs. In practice, mixtures of spices, herbs and oils that contain thymol, carvacrol and vanillin were used in piglet production. Among the phytogenic additives, extracts from agrimonia have been reported as eligible candidates that possess antioxidative, anti-inflammatory and antibacterial properties, and exert beneficial effects on lipid metabolism [5–8]. Agrimonia belongs to the family of Rosaceae and is a genus of 12–15 species of perennial herbaceous flowering plants. The most common species of agrimonia in Europe (*Agrimonia eupatoria* L. and *Agrimonia procera* Wallr.) are used in folk medicine traditionally for the treatment of diarrhea, colitis, grumbling appendix, cystitis and wound healing [9–11]. The major bioactive compounds of *Agrimonia eupatoria* (AE) are polyphenols such as flavonoids, mainly glycosides of luteolin and apigenin, and agrimoniin [12]. Recent data show that extracts from AE are capable of improving the antioxidative status of broiler chickens [13]. *Agrimonia procera* (AP) is very similar to AE [14], but seems to have a greater antioxidative capacity [15], and is characterized by a higher yield than the commonly used AE. The effects of AP in piglet nutrition and on cytokine synthesis in challenged porcine immune cells are currently unknown. The first objective of this study was to test the effects of AP on growth performance and diarrhea frequency of weaned piglets in two feeding experiments.

Weaning of piglets is often linked to a transient increase in inflammatory cytokines such as interleukin (IL)-1β, IL-6 and tumor necrosis factor (TNF)α [16] which are produced by monocytes and macrophages, respectively. These cytokines stimulate systemic effects of inflammation such as fever, loss of appetite, and mobilization of protein and fat. Conversely, IL-10 prevents excessive TNFα production [17–19]. The second objective of the study was to investigate putative effects of AP on the cytokine profile of lipopolysaccharides (LPS) challenged peripheral blood mononuclear cells (PBMC), isolated from healthy piglets.

## Results

### Composition of *Agrimonia procera*

One gram dried powder of the AP leaves that was used in experiment 1 contained 1.21 mg quercetin equivalents and 74 mg gallic acid equivalents. The dried powder of AP leaves and stalks which was used in experiment 2 contained 0.43 mg quercetin equivalents and 40 mg gallic acid equivalents per g. Agrimoniin and the luteolin and apigenin glycosides were identified as the main phytochemicals of AP. The AP powder from leaves contained 12.7 mg and the AP powder from leaves and stalks contained 6.38 mg agrimoniin per g of dry mass, respectively.

### Animal performance

During the feeding experiments all piglets remained healthy and did not need any medication. Mean initial and final body weights, daily weight gain, food intake and the food conversion ratio (FCR) are summarized in Tables 1 and 2. In both experiments, the final body weights and the daily weight gain were not influenced by AP (Tables 1 and 2).

**Table 1:** **Performance of weaned piglets fed diets without or with** ***Agrimonia procera***

**Parameter**	**Control**	***Agrimonia procera*** **(0.56 g/kg)**	**P-value**
Initial body weight (kg)	8.1 (0.7)	8.1 (0.8)	0.909
Final body weight (kg)	25.2 (2.8)	25.9 (3.1)	0.177
Daily weight gain (g/d)	407 (68)	425 (66)	0.148
Food intake (g/d)^1^	594 (63)	624 (63)	0.088
Food conversion ratio (kg/kg)^1^	1.46 (0.11)	1.46 (0.11)	0.972
Nitrogen intake (g/d)^2^	14.49 (1.33)	15.65 (0.32)	0.094
Nitrogen excretion^2^			
Feces (g/d)	2.99 (0.53)	3.31 (0.46)	0.340
Urine (g/d)	2.14 (0.34)	2.09 (0.37)	0.839
Retention (g/d)^2^	9.36 (0.66)	10.26 (0.51)*	0.044
Retention (%)^2^	64.8 (2.4)	65.6 (4.1)	0.712

Mean values (SD), n = 60 per group.

^1^Means of two piglets per pen were averaged.

^2^n = 5 per group.

*Significantly different from control (Student’s *t* test).

**Table 2:** **Performance of weaned piglets fed two different dosages of** ***Agrimonia procera***

**Parameter**	**Control**	***Agrimonia procera*** **(AP)**		
		**Low AP (0.87 g/kg)**	**High AP (8.7 g/kg)**	**P-value**
Initial body weight (kg)	8.4 (0.6)	8.3 (0.8)	8.6 (0.7)	0.346
Final body weight (kg)	23.1 (2.9)	24.1 (2.0)	23.4 (2.5)	0.445
Daily weight gain (g/d)	349 (61)	377 (35)	352 (46)	0.168
Food intake (g/d)	537 (79)	551 (54)	560 (58)	0.524
Food conversion ratio (kg/kg)	1.54 (0.08)^b^	1.46 (0.04)^c^	1.60 (0.08)^a^	<0.001

Mean values (SD), n = 20 per group.

^a,b,c^Values not sharing the same superscript are significantly different (Fisher’s test).

In experiment 1, the food and nitrogen intake tended to be higher in piglets fed the AP diets than in piglets fed the control diet (P < 0.10), whereas the FCR and the nitrogen excretion via urine and feces remained unaffected by AP treatment (Table 1). Collectively, piglets treated with AP showed a higher retention of nitrogen than the control piglets (Table 1).

In experiment 2, the food intake did not differ between the three groups of piglets, but piglets fed a diet with 0.87 g AP/kg exhibit an improved FCR compared to piglets fed none or 8.7 g AP per kg diet (Table 2). The consistency of feces was not different between the three groups of piglets (Table 3). The plasma activities of alanine aminotransferase (ALT) and aspartate aminotransferase (AST), indicative for liver damage, were not different between the AP groups and the control group (Table 4). The total antioxidant capacity of the plasma, analyzed by the Trolox equivalent antioxidant capacity assay, was not different between the three groups of piglets (Table 4).

**Table 3:** **Feces dry matter of weaned piglets fed two different dosages of** ***Agrimonia procera***

		***Agrimonia procera*** **(AP)**	
**Parameter**	**Control**	**Low AP (0.87 g/kg)**	**High AP (8.7 g/kg)**
Feces dry matter (%)			
1. Week	23.2 (6.0)	22.9 (4.1)	20.2 (7.1)
2. Week	25.9 (4.1)	26.5 (3.8)	24.0 (5.2)
3. Week	27.1 (2.7)	27.4 (2.3)	27.0 (2.7)

Mean values (SD), n = 20 per group.

**Table 4:** **Aminotransferase activities and total antioxidant capacity in plasma of weaned piglets fed two different dosages of** ***Agrimonia procera***

		***Agrimonia procera*** **(AP)**	
**Parameter**	**Control**	**Low AP (0.87 g/kg)**	**High AP (8.7 g/kg)**
ALT^1^ (U/l)	18.7 (4.8)	17.3 (4.0)	18.1 (4.6)
AST (U/l)	40.8 (10.3)	41.6 (15.3)	46.5 (11.0)
TEAC (μmol/l)	7.60 (0.63)	7.63 (1.06)	7.67 (0.77)

Mean values (SD), n = 20 per group.

^1^ALT: alanine aminotransferase; AST: aspartate aminotransferase; TEAC: Trolox equivalent antioxidant capacity.

### Cytokine mRNA expression and TNFα release of PBMC in response to AP extract

**Figure 1:**
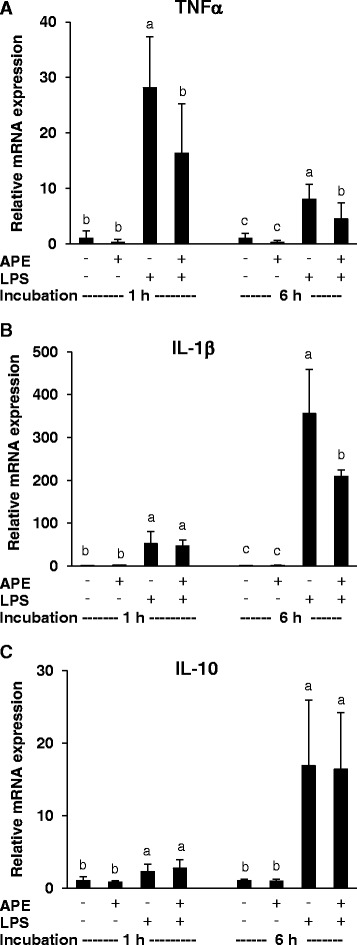
**Effects of** ***Agrimonia procera*** **extract (APE) on cytokine mRNA expression by porcine PBMC.** Relative mRNA concentrations of TNFα **(A)**, IL-1β **(B)** and IL-10 **(C)** were analyzed in non-stimulated and LPS (1 μg/ml)-stimulated PBMC (2 × 10^6^ per ml RPMI 1640 medium) treated with 0 or 0.1% APE for 1 and 6 h, respectively. PBMC were isolated from 4 healthy piglets. mRNA expression was normalized to that of β-actin. Data represent mean ± SD (n = 4). ^a,b^Bars with different superscript letters within an incubation period differ significantly (P < 0.05, Fisher’s test).

PBMC challenged with LPS for 1 and 6 h, respectively, revealed a markedly higher mRNA expression of TNFα, IL-1β and IL-10 than non-stimulated PBMC (P < 0.05). The mRNA level of TNFα was highest after 1 h, whereas the mRNA levels of IL-1β and IL-10 where highest after 6 h of LPS stimulation (Figure 1). In non-stimulated PBMC, AP extract (APE) revealed no significant effect on the mRNA expression of the cytokines (Figure 1). LPS-challenged PBMC treated for 1 and 6 h with 0.1% (v/v) of APE revealed a lower TNFα mRNA expression than cells without APE treatment (P < 0.05, Figure 1A). The mRNA expression of IL-1β was also reduced in response to treatment with 0.1% of APE, but this effect was only seen after 6 h (P < 0.05), but not after1 h. The mRNA expression of the anti-inflammatory IL-10 was not affected by APE (Figure 1).

**Figure 2:**
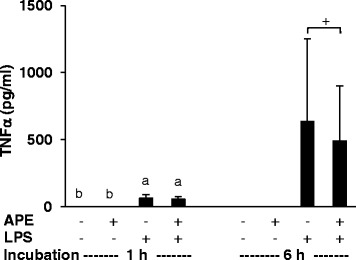
**Effect of** ***Agrimonia procera*** **extract (APE) on TNF** **α** **production by porcine PBMC.** The TNFα concentration was analyzed in culture supernatant of non-stimulated and LPS (1 μg/ml)-stimulated PBMC (2 × 10^6^ per ml RPMI 1640 medium) treated with 0 or 0.1% APE for 1 and 6 h, respectively. PBMC were isolated from 4 healthy piglets. TNFα was determined using an ELISA. Data represent mean ± SD (n = 4). ^a,b^ Bars with different superscript letters within an incubation period differ significantly (P < 0.05, Fisher’s test). ^+^ tended to be different (P = 0.067, Student’s *t* test).

To test whether the altered TNFα mRNA expression in response to LPS and APE was associated with an altered protein expression and release of TNFα, we analyzed the TNFα concentration in the cell media. The incubation media of non-stimulated PBMC was characterized by very low TNFα concentrations (Figure 2). LPS stimulation increased the extracellular TNFα concentration, whereby the increase was much higher after 6 h than after 1 h of incubation (P < 0.05; Figure 2). Treatment of LPS-challenged PMBC with 0.1% APE for 1 h had no effect on the extracellular TNFα concentration. However, PBMC treated with APE for 6 h tended to release less TNFα into the cell media than those without APE treatment (P = 0.067).

**Figure 3:**
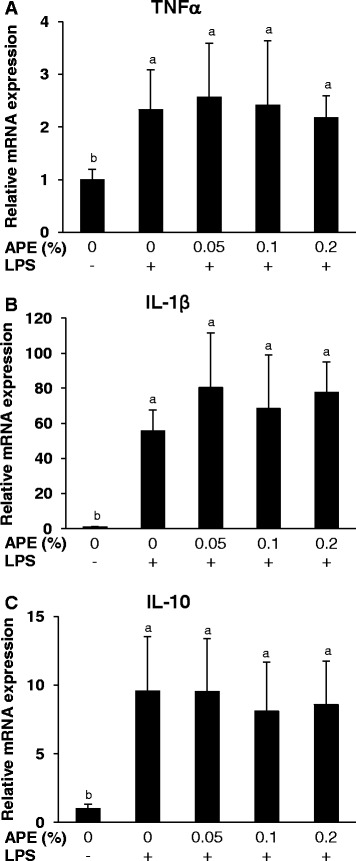
**Effect of** ***Agrimonia procera*** **extract (APE) on cytokine mRNA expression by porcine PBMC.** Relative mRNA concentrations of TNFα **(A)**, IL-1β **(B)** and IL-10 **(C)** were analyzed in non-stimulated and LPS (1 μg/ml)-stimulated PBMC (2 × 10^6^ per ml RPMI 1640 medium) treated with 0, 0.05, 0.1 or 0.2% APE for 20 h. PBMC were isolated from 6 healthy piglets. The relative mRNA expression was normalized to that of RPP0 and SDHA. Data represent mean ± SD (n = 6). ^a,b^Bars with different superscript letters differ significantly (P < 0.05, Fisher’s test).

**Figure 4:**
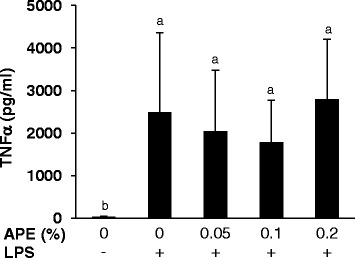
**Effect of** ***Agrimonia procera*** **extract (APE) on TNF** **α** **production by porcine PBMC.** The TNFα concentration was analyzed in culture supernatant of LPS (1 μg/ml)-stimulated PBMC (2 × 10^6^ per ml RPMI 1640 medium) treated with 0, 0.05, 0.1 or 0.2% APE for 20 h. Non-stimulated cells without APE treatment served as control. PBMC were isolated from 6 healthy piglets. The amount of TNFα was determined using an ELISA. Data represent mean ± SD (n = 6). ^a,b^Bars with different superscript letters differ significantly (P < 0.05, Fisher’s test).

Data from the second in vitro experiment showed that PBMC challenged for 20 h with LPS had higher mRNA abundances of TNFα, IL-1β and IL-10 than non-stimulated PBMC (P < 0.05, Figure 3). Treatment of cells with increasing dosages of APE for 20 h had no effect on the mRNA expression of these cytokines (Figure 3), and also the extracellular TNFα concentration was not influenced by those treatments (Figure 4).

## Discussion

It is suggested that phytochemicals are attributable to the proposed beneficial health effects of herbal feed additives in livestock. Analyses from the current study show that AP belongs to plants that are rich in flavonoids and polyphenols, especially in agrimoniin which is a major bitter plant polyphenolic compound of AP. By comparison of AP with other agrimonia species, for example those which were used for humans, AP contained less total flavonoids than AE, although the total polyphenols of AP are higher compared to the extracts from flowering aerial parts of other agrimonia species [15]. However, the differences in contents of phytochemicals between agrimonia species and within a defined species could be also caused by differences in the harvest period, the growth conditions and the plant parts that are used for the analysis.

Recent studies have documented that herbal feed additives could improve the performance of piglets, including food intake [20,21]. A trend towards a higher food intake was also seen in the AP treated piglets from experiment 1, assuming that also herbals such as AP, may stimulate the appetite of piglets. Although such an appetite-stimulating effect of AP was not seen in experiment 2, piglets that were fed a diet with 0.87 g AP per kg diet revealed a lower FCR than piglets treated without AP. Both effects of AP on food intake and FCR are desirable and indicative for an improved performance. The fact that piglets that were fed the high AP diet containing 8.7 g AP per kg diet revealed a deteriorated FCR compared to that of control piglets suggests a biphasic effect of AP with optimum effects in the low dosage range. Aminotransferase activities in plasma which are sensitive markers of possible tissue damage, particularly liver toxicity, do not imply a toxic effect of the high AP diet, because circulating ALT and AST activities were not different between the AP groups and the control group. Weaning is most commonly associated with an increased proliferation of gut microbiota and hypersecretion of bacterial enterotoxins [22]. Non-absorbed dietary phenolics and their metabolites have been assumed to exert significant effects on the intestinal environment by modulation of the microbiota [23,24]. It is possible that the beneficial effect of the low AP diet on FCR and nitrogen retention in our study was caused by beneficial effects on gut microbiota. Antioxidative effects of AP appear to play no role, because AP fed piglets revealed no higher antioxidant capacity of the plasma than the control animals. On the other hand the adverse effect of the high AP diet on FCR could possibly be provoked by the high amounts of tannins such as agrimoniin which are known to deteriorate the digestibility of nutrients, in particular that of protein [25].

Various studies demonstrated the anti-inflammatory potential of agrimonia [26,7]. Although the mechanisms of the antiinflammatory effects of plant extracts have not yet been fully elucidated, recent data show that extracts from AE were capable of reducing the activity of nuclear factor-kappa-light-chain-enhancer of activated B cells (NF-κB) via suppression of its nuclear translocation [27]. It is discussed that polyphenols activate heterodimers of NF-E2-related factors 2/antioxidant responsive element pathway which in turn may modify the NF-κB activity [28,29]. In order to test the anti-inflammatory potential of AP, we used therefore LPS-challenged PBMC from healthy piglets as inflammation model. LPS are found in the outer membrane of gram-negative bacteria, where they function as endotoxins and elicit strong immune responses. We found that extracts from AP were capable of reducing the mRNA expression of pro-inflammatory cytokines such as TNFα and IL-1β in the LPS-challenged PBMC, without affecting the mRNA expression of the anti-inflammatory IL-10. This means that AP is capable of changing the cytokine profile towards a reduction of the pro-inflammatory response under LPS-challenging conditions. Moreover, the data show a time-dependent impact of APE on the mRNA expression of pro-inflammatory cytokines, with strong effects during the first hours, and failing effects after 20 h. Pro-inflammatory cytokines and their receptors are known to antagonize the anabolic effects of insulin-like growth factor (IGF) 1 by reducing its synthesis and by a reduction of the responsiveness of target tissue to IGF-1 stimulation [30]. Considering the fact that weaning-associated intestinal inflammation [16] is usually linked to adverse effects on nutrient metabolism, growth, and muscle development in pigs [31], a reduction of a severe pro-inflammation state by AP could contribute to reduce acute symptoms of infection and to maintain normal growth conditions. We assume that the phytochemicals of AP are responsible for the down regulation of the pro-inflammatory cytokines since apigenin and luteolin has been already identified as inhibitors of pro-inflammatory cytokine production in LPS-stimulated PBMC [32], and as factors which reduce the transcriptional activity of NF-κB in LPS-stimulated primary monocytes [33] and macrophages [34].

## Conclusion

Collectively, the data show that low dosages of AP could provide beneficial effects on feed intake and FCR in piglets. Data from in vitro studies with LPS-challenged PBMC indicate that AP extracts are capable of reducing the mRNA expression and synthesis of pro-inflammatory cytokines. The actual efficacy of AP to maintain the health of piglets and its potential to reduce the application of antimicrobials has to be investigated under practical animal production conditions.

## Methods

### Animal experiments

All experimental procedures described were approved by the local Animal Care and Use Committee of the council of Saxony-Anhalt (approval number: 42502 Merbitz-15MLU) and were in accordance with German animal welfare legislation. We declare that we adhered to the ARRIVE guidelines (see Additional file 1).

### Animals and diets

Two studies were performed with 120 (experiment 1) and 60 (experiment 2) healthy 4-week old weaned hybrid piglets [(German Landrace × German Edelschwein) × Piétrain]. The piglets were purchased from a local breeder. For the first feeding experiment, castrated male and female (1:1) piglets were randomly assigned to 2 groups of 60 each. One group received a basal diet with 0 g AP per kg diet (control), the other group received a diet with 0.56 g AP per kg diet as finely ground AP leaves (Exsemine GmbH, Zappendorf, Germany). To test whether higher amounts of AP are more effective in influencing the performance of the piglets, a second feeding experiment was conducted. In this experiment, castrated male and female (1:1) piglets were randomly assigned to 3 groups of 20 each, and either received 0, 0.87 and 8.7 g AP per kg diet, respectively, with their diet as finely ground AP leaves and stalks. The amounts of AP were chosen to attain an equal concentration of total polyphenol as in experiment 1 (35 mg per g AP) in the low AP diet and a 10 times higher concentration (350 mg per g AP) in the high AP diet.

The experimental diets were fed for a total of 6 weeks. In the first two weeks a pre-starter diet served as basal diet and in the following 4 weeks the piglets received standard starter diets (Table 5). The AP powder was added at the expense of the basal diet. The basal diets were formulated to meet the nutrient requirements recommended for piglets [35]. All piglets had free access to food. Water was available ad libitum from a nipple drinker system during the whole experiment. The piglets were housed in flat deck pens (two piglets per pen in experiment 1 and one piglet per pen in experiment 2). All animals were kept in an environmentally controlled facility with a temperature of 29°C at the beginning which decreased to 22°C at the end of the experiments, relative humidity between 55-60%, and light from 6:00 am to 6:00 pm.

**Table 5:** **Composition of the basal diets (g/kg)**

**Ingredients**	**Pre-starter** ^**1**^	**Starter** ^**2**^
Wheat	40.0	40.0
Barley	19.45	20.0
Maize	10.0	12.0
Soybean meal (48% crude protein)	18.0	18.42
Whey powder	5.0	2.5
Wheat semolina bran	3.0	3.0
Soybean oil	2.5	2.4
Mono-calcium-phosphate	0.5	0.5
Lysine HCl	0.68	0.55
DL-methionine	0.27	0.2
L-threonine	0.3	0.23
L-tryptophan	0.08	0.06
L-valine	0.22	0.14
Mineral and vitamin premix^3^	2.0	2.0

^1^based on 13.9 MJ net energy (NE)/kg and standardized ileal digestible amino acids [lysine 12 g/kg, methionine/cystine 6.83 g/kg, threonine 7.42 g/kg, tryptophan 2.4 g/kg].

^2^based on 13.77 MJ NE/kg and standardized ileal digestible amino acids [lysine 10.1 g/kg, methionine/cystine 6.15 g/kg, threonine 6.59 g/kg, tryptophan 2.16 g/kg].

^3^per kg premix: calcium 265 g, sodium 60 g, phosphorus 40 g, magnesium 5.5 g, iron 2.85 g, zinc 2.2 g, copper 750 mg, manganese 730 mg, cobalt 15 mg, iodine 10 mg, selenium 10 mg, vitamin A 1,000,000 IU, vitamin D 100,000 IU, nicotinic acid 2.6 g, vitamin E 2 g, pantothenic acid 1.25 g, riboflavin 500 mg, pyridoxine 300 mg, thiamin 200 mg, vitamin K 150 mg, folic acid 30 mg, vitamin B_12_ 2 mg, biotin 1 mg, choline chloride 20 g, flavoring substance 1.5 g.

Food intake and body weight were recorded weekly. Based on these data, average daily weight gain, daily food intake and FCR (food intake/weight gain) were calculated. At day 28 of the experiment 1, five castrated male piglets per group were transferred to metabolic cages for a total of 5 days to estimate the nitrogen retention. During this period the piglets were fed controlled amounts of the experimental diets twice a day (07:30 am and 03:00 pm). After a 24 h adaptation period feces and urine were collected quantitatively. In experiment 2, the feces consistency was analyzed once a week in the first three weeks of the study by analyzing the dry matter content of the feces. At day 28, blood was collected from the vena jugularis for analysis of plasma aminotransferases and TEAC.

### Analysis of the phytochemicals in AP

For phytochemical analysis, the AP leaves and stalks were crushed and homogenized. The total polyphenol content defined as gallic acid equivalent and the total flavonoid content defined as quercetin equivalent were analyzed according to [15] by using Folin-Ciocalteu phenol reagent and aluminum chloride hexahydrate (both Sigma-Aldrich, Sternheim, Germany), respectively.

The individual compounds of AP were determined in cooperation with the Department of Pharmacognosy and Molecular Basis of Phytotherapy, Medical University of Warsaw. The plant compounds were extracted with hot deionized water and analyzed with UHPLC-DAD-MS as described previously [12]. The HPLC analyses were performed using an Ultimate 3000 series system (Dionex, Idtsein, Germany), and a UV–vis in the range of 200–450 nm and MS spectra.

### Nitrogen analyses

Nitrogen concentrations of diets, feces and urine were analyzed by the Kjeltec system (Kjeltec 2300, Foss, Hamburg, Germany). The dry matter content of the diets and feces was analyzed by drying of the samples at 105°C for 4 h.

### Analysis of plasma aminotransferases and the antioxidant capacity

To monitor any adverse or toxic effects of the AP diets, we analyzed the plasma activities of alanine aminotransferase (ALT) and aspartate aminotransferase (AST) that are sensitive markers of possible tissue damage. Both enzymes were photometrically determined by use of commercial test kits (DiaSys Diagnostic Systems GmbH, Holzheim, Germany).

To reveal the antioxidant capacity of plasma in response to the AP diets, a Trolox equivalent antioxidant capacity (TEAC) assay was used. TEAC was measured according the method of Wang et al. [36] and Mueller et al. [37]. The method is based on the suppression of the absorbance of the 2,20-azino-bis (3-ethylbenzothiazoline-6-sulphonic acid (ABTS) radical formation by antioxidants in the test samples. The reaction mixture contained PBS buffer, ABTS reagent (0.15 mM), H_2_O_2_ (0.1 mM) and metmyoglobin (2.52 mM). The absorbance was measured at 600 nm for 15 min at 20°C. Sample values were calculated using a standard curve and were expressed as μmol/l.

### In vitro experiments

#### Isolation of the PBMC

To investigate the effects of AP on the expression of cytokines, porcine PBMC were obtained from healthy weaned piglets (mean body weight 18 kg). For the first experiment, 4 piglets were used as donors, and for the second experiment, PBMC of 6 piglets were included. The cells were isolated from peripheral blood drawn from the anterior vena cava by density gradient separation with Histopaque®-1077 (Sigma-Aldrich) according to the manufacturer’s protocol. In brief, whole EDTA-blood was layered (1:1) onto Histopaque. After centrifugation at 400 × g for 30 min at room temperature, the mononuclear cells at the interface were harvested, washed three times with phosphate buffered saline solution and re-suspended in RPMI 1640 medium (Life Technologies, Darmstadt, Germany) supplemented with 5% heat-inactivated fetal bovine serum (FBS, Life Technologies).

### Cell treatments

For the incubation experiments, an ethanolic extract of AP was prepared by dissolving 200 mg of AP powder in 1 ml of ethanol (99% pure). The mixture was incubated for 30 min at 75°C. The insoluble constituents were removed by centrifugation at 2,500 × g for 5 min and the resulting supernatant was stored at −20°C until further use.

In both experiments, the PBMC were seeded into 24-multiwell plates at a density of 2 × 10^6^ cells per ml RPMI 1640 medium. In the first cell experiment, the PBMC were incubated in RPMI 1640 medium without or with 1 μg/ml LPS (Escherichia coli 0127:B8, Sigma-Aldrich), and with 0 or 0.1% (v/v) of APE for 1 and 6 h, respectively, at 37°C and 5% CO_2_. In the second cell experiment, LPS (1 μg/ml)-challenged PBMC were treated with 0, 0.05, 0.1 or 0.2% of APE for 20 h. The longer incubation time in experiment 2 was used to allow enough time for the synthesis of TNFα and to get significant TNFα amount to be performed. Cells without LPS stimulation and 0% APE in the incubation medium served as controls. APE was added to the culture medium in a maximal volume (<0.4%) and the same volume of ethanol as the solvent was added to the medium of the control cells. Subsequent to the treatment PBMC were transferred into tubes, harvested by centrifugation at 900 × g for 2 min and used for analysis of mRNA concentrations of TNFα, IL-1β and IL-10 by real-time detection RT-PCR. The culture supernatant was centrifuged a second time at 13,000 × g for 5 min to remove cell debris and was stored at −20°C until quantification of secreted TNFα by means of an ELISA.

### Real-time RT-PCR analysis

Total RNA was isolated from PBMC using peqGOLD TriFast™ (Peqlab, Erlangen, Germany) according to the manufacturer’s protocol. RNA concentration and purity were estimated from the optical density at 260 and 280 nm, respectively. 0.4 μg of total RNA was subjected to cDNA synthesis at 42°C for 60 min using M-MuLV Reverse Transcriptase (MBI Fermentas, St. Leon-Rot, Germany) and oligo dT18-primer (Eurofins MWG Operon, Ebersberg, Germany). For determination of mRNA expression levels real-time detection PCR using the Rotorgene 6000 system (Corbett Research, Mortlake, Australia) was applied. Real-time PCR was performed by using 1.25 U GoTaq Flexi DNA polymerase (Promega, Mannheim, Germany), 500 μM dNTP (Ares Bioscience, Cologne, Germany), SYBR® Green I (Sigma, Taufkirchen, Germany) and 26.7 pmol of the specific primers (Eurofins MWG Operon). Each PCR cycle comprised denaturation for 20 s at 95°C, annealing for 35 s at primer-specific temperature (57-64°C) and elongation for 55 s at 72°C. For determination of the mRNA concentration, threshold cycle (C_T_) and amplification efficiency were obtained from each amplification curve using the RotorGene 1.7 software (Corbett Research). Calculation of the relative mRNA concentration was made using the method previously described by Pfaffl [38]. The amplification data of single genes were normalized to the expression of stable housekeeping genes. In the first study the most stable reference gene was β-actin; in the second study, succinate dehydrogenase complex subunit A (SDHA) and ribosomal phosphoprotein large PO subunit (RPP0) were used as reference genes because both genes had proved to be the most stable genes. The relative mRNA concentrations were expressed as fold-changes relative to the control. The primer sequences and characteristics are shown in Table 6.

**Table 6:** **Characteristics of the primers used in RT-PCR analysis and PCR product sizes**

**Gene**	**Forward primer (from 5′ to 3′)**	**Product size (bp)**	**NCBI GenBank number**
	**Reverse primer (from 5′to 3′)**		
β-actin	GACATCCGCAAGGACCTCTA	205	DQ_845171.1
	ACATCTGCTGGAAGGTGGAC		
SDHA^1^	CTAGCCCCCGTCGCAAAGG	380	DQ_402993
	AGTTTGCCCCCAGGCGGTTG		
RPP0	CAACCCTGAAGTGCTTGACA	204	NM_001098598.1
	GCCTTGACCTTTTCAGCAAG		
TNFα	AACCCTCTGGCCCAAGGA	57	NM_214022.1
	GGCGACGGGCTTATCTGA		
IL-1β	AAAGGGGACTTGAAGAGAG	286	NM_001005149.1
	CTGCTTGAGAGGTGCTGATGT		
IL-10	GCATCCACTTCCCAACCA	446	NM_214041.1
	CTTCCTCATCTTCATCGTCAT		

^1^SDHA, succinate dehydrogenase complex subunit A; RPP0, ribosomal phosphoprotein large PO subunit; TNF, tumor necrosis factor; IL, interleukin.

### Analysis of TNFα in cell culture supernatant

The concentration of TNFα in the supernatant was determined by a porcine TNFα ELISA kit (R&D Systems, Abingdon, UK) according to the manufacturer’s instructions.

### Statistical analysis

Statistical analysis was carried out by using one way ANOVA (Minitab, Version 13, State College, PA, USA). In the case of significant F-values (P < 0.05) comparisons of more than two groups were done using the Fisher’s test. The Student’s *t* test was used for the comparison of two groups. Means were considered significantly different at P < 0.05. Data represent means ± standard deviation (SD).

## Additional file

Additional file 1:
**The ARRIVE Guidelines Checklist.**

## Abbreviations

AE: *Agrimonia eupatoria*
AP: *Agrimonia procera*
APE: *Agrimonia procera* extract
ALT: Alanine aminotransferase
AST: Aspartate aminotransferase
DM: Dry matter
FCR: Food conversion ratio
IGF: Insulin-like growth factor
IκB: Inhibitor protein kappa B
IL: Interleukin
LPS: Lipopolysaccharide
NF-κB: Nuclear factor-kappaB
PBMC: Peripheral blood mononuclear cells
RPP0: Ribosomal phosphoprotein large PO subunit
SDHA: Succinate dehydrogenase complex subunit A
TNF: Tumor necrosis factor
TEAC: Trolox equivalent antioxidant capacity
